# dbPSHP: a database of recent positive selection across human populations

**DOI:** 10.1093/nar/gkt1052

**Published:** 2013-11-03

**Authors:** Mulin Jun Li, Lily Yan Wang, Zhengyuan Xia, Maria P. Wong, Pak Chung Sham, Junwen Wang

**Affiliations:** ^1^Department of Biochemistry, LKS Faculty of Medicine, The University of Hong Kong, Hong Kong SAR, China, ^2^Shenzhen Institute of Research and Innovation, The University of Hong Kong, Shenzhen, Guangdong 518057, China, ^3^Department of Anaesthesiology, LKS Faculty of Medicine, The University of Hong Kong, Hong Kong SAR, China, ^4^Department of Pathology, LKS Faculty of Medicine, The University of Hong Kong, Hong Kong SAR, China, ^5^Department of Psychiatry, LKS Faculty of Medicine, The University of Hong Kong, Hong Kong SAR, China, ^6^State Key Laboratory in Cognitive and Brain Sciences, The University of Hong Kong, Hong Kong SAR, China and ^7^Centre for Genomic Sciences, LKS Faculty of Medicine, The University of Hong Kong, Hong Kong SAR, China

## Abstract

The dbPSHP database (http://jjwanglab.org/dbpshp) aims to help researchers to efficiently identify, validate and visualize putative positively selected loci in human evolution and further discover the mechanism governing these natural selections. Recent evolution of human populations at the genomic level reflects the adaptations to the living environments, including climate change and availability and stability of nutrients. Many genetic regions under positive selection have been identified, which assist us to understand how natural selection has shaped population differences. Here, we manually collect recent positive selections in different human populations, consisting of 15 472 loci from 132 publications. We further compiled a database that used 15 statistical terms of different evolutionary attributes for single nucleotide variant sites from the HapMap 3 and 1000 Genomes Project to identify putative regions under positive selection. These attributes include variant allele/genotype properties, variant heterozygosity, within population diversity, long-range haplotypes, pairwise population differentiation and evolutionary conservation. We also provide interactive pages for visualization and annotation of different selective signals. The database is freely available to the public and will be frequently updated.

## INTRODUCTION

Natural selection plays a crucial role in the evolution of species, where random mutations are undergoing positive, purifying or balancing selection (1) for adaptation to the living environments including climate change, availability and stability of nutrients, introduction of novel disease agents, dispersed niche, etc. Recent evolutionary adaptations in the human lineage have been reflected by many population-specific traits such as pigmentation, malaria resistance and lactose tolerance (2–4). Many genetic regions of human genome under positive selection have been successfully identified, which assist us in understanding how natural selection has shaped population differences (5). Signatures of selection can be detected by observing the underlying patterns of DNA polymorphisms in one or different populations, which will facilitate the identification of positively selected genes or loci that are associated with specific function, trait or disease (6,7).

Statistical methods and tools have been successfully developed to detect genome-wide selective signals based on genetic data of human populations. Given one population, positive or negative selection tends to skew the allele frequencies comparing with neutral model. Statistics such as Tajima’s D (8) and Fay and Wu’s H (9) can detect a locus’s departures from neutrality and underlying selection. Linkage information can also be used to infer the selection signals. Besides, strong selection signal can also be discovered by searching a long-range haplotype. Extended haplotype homozygosity (EHH) (10) and integrated haplotype score (iHS) (11) have been used to capture these loci based on the length of haplotypes associated with a given allele. Recently, several new programs, such as HaploPS and SweeD, have been developed to efficiently search the regions on the genome carrying positive selection signals with higher sensitivity and specificity (12,13). Positive selection can also be identified by tracking the increment of identity-by-descent among individuals in a population (14,15). Moreover, large allele frequency differences between populations can be measured by fixation index (*F*_ST_) (16) at each single nucleotide polymorphism (SNP) locus in the genome. Researchers also developed a tool, cross-population extended haplotype homozygosity test (XP-EHH), to detect ongoing or nearly fixed selective sweeps by comparing haplotypes from two populations (17). The cross-population composite likelihood ratio test (XP-CLR) scans multi-locus allele frequency differentiation between two populations to detect selective sweeps in analogy to EHH (18). Last, rejected substitution is adopted in genomic evolutionary rate profiling (19) to assess the strength of the selected elements on single nucleotide level.

The causal mutations for population adaptation have been proved to locate in many functional loci on the human genome. For different human populations, studies have shown that environmental changes, such as diet, climate and infectious disease, have caused advantageous rapid amino acid evolutions and consequently affect protein functions (20). Analysis has also been performed to identify a number of positively selected synonymous variants affecting the translation efficiency (21). Recently, researchers revealed that local adaptations have a higher chance to affect gene expression than amino acid sequence by studying selective signals between gene expression-associated SNPs and nonsynonymous SNPs (22). Until now, over hundreds of function-associated regions/genes have been reportedly undergoing positive selection from different human populations by inferring population genetic data. However, it is a tedious and time-consuming process of curation if researchers want to retrieve the selection information of their regions of interest or traits from literature. By far, little resources are available for users to search for known selective regions and their associated function effects.

However, the selective signals detected by aforementioned statistical methods are not always consistent in terms of the degree of derived allele frequency, which is usually varied by different datasets. To accurately identify true positive selection and the causal mutation, we need to combine different statistical values. A composite of multiple signals method has been proposed to combine five selective signals with satisfactory power (23). Some resources such as SNP@Ethnos (24), Haplotter (11), SNP@Evolution (25) and dbCLINE (26) have also provided respective selection signals for some populations in early HapMap dataset. However, more supporting signals are needed for explicit elaboration, and more world-wide populations should be investigated based on larger sample size. The recent International HapMap Project and 1000 Genomes Project have produced high quality genotyping data in a large sample size of different human populations, which enable us to systematically detect natural selection signals in a genome wide scale (27,28). Therefore, a comprehensive, easy-to-use and up-to-date resource focusing on recent human positive selection is urgently required.

Here we developed a database dbPSHP, a user friendly web portal on recent positive selection across human populations. We first manually collected 15 472 recent positive selections and related information in different human populations from literature. We further compiled a database that contains 15 calculated statistical signals for SNP sites from the HapMap 3 and 1000 Genomes Projects, which focus on variant allele/genotype properties, variant heterozygosity, within population diversity, long-range haplotypes, pairwise population differentiation and evolutionary conservation. We also provided interactive pages for visualization and annotation of different selective signals.

## DATABASE DESIGN AND CONTENT

dbPSHP provides a manually curated dataset of positively selected loci of human populations from literature. It also consists of a variety of important attributes associated with recent human selection for one or pairwise populations under a consistent framework. The selection signals are evaluated on several aspects including ancestral and derived allele, allele frequency, genotype frequency, Hardy–Weinberg equilibrium (HWE), heterozygosity, nucleotide diversity, Tajima’s D, iHH, iHS, derived allele frequency difference (ΔDAF), fixation index (*F*_ST_), XP-EHH, XP-CLR, neutral rate, and rejected substitution (Table 1). Furthermore, dbPSHP has been designed as a knowledge base and web service that offers a rapid search and interactive interface for the users.

**Table 1.** The scope and calculated scores in the dbPSHP database

Attribute	Evaluation term	Abbreviation
Variant genotype properties	Derived allele	DA
	Ancestral allele	AA
	Allele frequency	DAF
		AAF
	Genotype frequency	GFHOM1
		GFHET
		GFHOM2
	Hardy–Weinberg equilibrium	HWE1
		HWE2
Variant heterozygosity	Heterozygosity	HET
	Nucleotide diversity	PI
Within population diversity	Tajima’s D	TD
Long-range haplotypes	Integrated extended haplotype homozygosity	IHH
	Integrated haplotype score	UIHS
		IHS
Differentiation between populations	Difference of derived allele frequency	DDAF
		DDAF_POP1_POP2
	Fixation index	FST1
		FST1_POP1_POP2
		FST2
		FST2_POP1_POP2
	Cross-population extended haplotype homozygosity	UXPEHH
		XPEHH_POP1_POP2
	Cross-population composite likelihood ratio	XPCLR
		XPCLR_POP1_POP2
Evolutionary conservation	Neutral rate	NR
	Rejected substitution	RS

DAF is the allele frequency for the derived allele; AAF is the allele frequency for the ancestral allele; GFHOM1 is the genotype frequency for homozygous derived allele AA; GFHET is the genotype frequency for heterozygous Aa; GFHOM2 is the genotype frequency for homozygous ancestral allele aa; HWE1 is the value of simple chi square goodness-of-fit test; HWE2 is the *P*-value of exact test; FST1 is the *F*_ST_ of Wright’s approximate formula; FST2 is the *F*_ST_ of Cockerham & Weir estimator; UIHS is the unstandardized integrated haplotype score; UXPEHH is the unstandardized cross-population extended haplotype homozygosity; POP1_POP2 represents the pairwise scores of two specific populations (Supplementary Methods).

We started with data collection from the publications attempting to study positively selected loci/genes related to specific functions/traits/diseases of human populations during recent human evolution. We manually searched these publications through PubMed and occasional collection of some specific reports by natural selection related keywords (details in Supplementary Methods). The current version of dbPSHP contains 15 472 manually collected loci/genes under positive selection from 132 publications. Among them, 101 publications attempt to study the specific adaptive traits, and 31 publications detect the genome-wide selective signals with different statistical methods.

We then processed the genetic data of different populations using the International HapMap phase 3 and the 1000 Genomes Project phase 1 (details in Supplementary Methods). We pre-computed statistical scores in different categories that mainly include variant allele/genotype frequency, variant heterozygosity, within population diversity, long-range haplotypes, pairwise population differentiation and evolutionary conservation (Supplementary Table S3 and Supplementary Methods).

There are different criteria to determine whether the investigated loci have been undergoing positive selection. High frequency of derived allele, deviations from HWE, reduced heterozygosity, negative Tajima's D, high *F*_ST_ value and relatively higher iHS more or less indicate the selective signals. To facilitate the identification of true signals, we designed a filtering function by a set of defined score cutoff, which have been frequently used as empirical estimation of positive selection in current evolution studies. We further generated a list of putatively causal mutations for each population using these hard filtering (Supplementary Methods).

## EVALUATION

To evaluate the reliability and accuracy of the statistical scores in dbPSHP, we first used two well-known cases under strong positive selection in specific population. Lactose tolerance has been previously identified as the positive selection in a large fraction of individuals of European descent after domestication of cattle, which genetically caused by a mutation in the lactase gene (*LCT*) (4). We validated the statistical scores for all of genetic variants in the *LCT* gene and nearby 500 kb genetic hitchhiking region in the CEU population. We found this positively selected region is significantly supported by all critical signals of most genetic variants in both HapMap 3 and 1000 Genomes Project dataset, including highly deviated derived allele frequency (ΔDAF), distinguished iHS and high *F*_ST_, XP-EHH and XP-CLR values compared with other populations (Supplementary Figures S1 and S2). Further, we used another well studied gene, *SLC24A5*, related to the selection of lighter pigmentation between Europeans and West Africans (29). We checked the selective scores along the *SLC24A5* and neighbouring selective sweep and we found, for CEU population of both HapMap 3 and 1000 Genomes Project dataset, there are increased signals of derived allele frequency and other indicators, especially in the downstream of *SLC24A5* gene (Supplementary Figures S3 and S4).

Furthermore, we measured the overall reliability of pre-calculated scores in dbPSHP by comparing the score distribution between reported selective region and background. We collected 997 CEU loci, 574 YRI loci and 516 CHB loci from our curated positive selection list. We then extracted all genetic variants within these regions from both HapMap 3 and 1000 Genomes Project dataset. We constructed background genetic variants by randomly selecting the same number of genomic regions. We performed Mann–Whitney U test, for *F*_ST_, |iHS|, |XP-EHH| and XP-CLR, to examine whether the selective scores in curated regions (regarded as under positive selection) are significantly larger than those in the background. We finally observed significant differences for almost all cases in different populations and the SNP dataset (Supplementary Table S4). The experiment further confirmed the usability of dbPSHP as a useful resource in the studies of recent human evolution.

Although there are some resources, such as SNP@Ethnos, Haplotter and SNP@Evolution, that selectively calculate particular selection scores in some populations using a different version of the HapMap dataset, it can hardly satisfy the immediate requirements of human evolutionary biology and population genetics. Even for the frequently used dataset CMS (23), it only provides five statistical scores (iHS, XP-EHH, ΔiHH, ΔDAF and *F*_ST_) on limited population, as well as a simple query interface. Comparing with these resources, dbPSHP systematically curates reported function-related regions/genes under recent positive selection in the human populations from literature. It also constructs a database integrating up to 15 statistical terms for positive selection by a large number of populations, latest human genetic dataset and interactive user interfaces, which allows detecting a different level of positive selections and facilitates better hypothesis generation (Supplementary Table S5).

## USAGE

dbPSHP website accepts three input formats including dbSNP ID, genomic locus and RefGene name. dbSNP ID will be converted to dbSNP 137 according to the SNP track history RsMergeArch. Genomic locus can be either a site (e.g. chr2:136575199) or a region (e.g. chr5:33944721-33984780). Both gene official symbol and Refseq accession number are supported as queries. For sanity visualization, the system will extend 50 kb surrounding regions if a user inputs a signal site. Users can also select the SNP data set (HapMap3 or 1000 Genomes Project) and investigated population in the input page. Also, a user can filter and sort the selection scores under different combination of empirical cutoffs.

**Figure 1.**
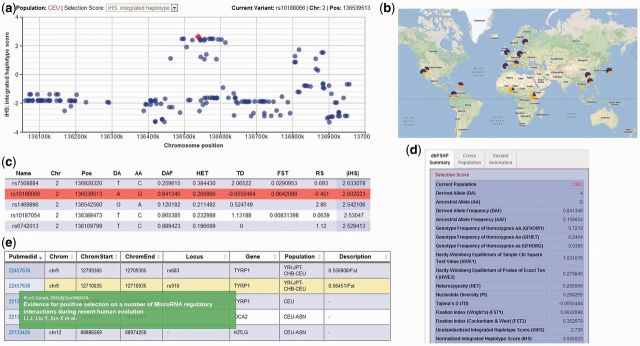
The main functional units of dbPSHP interface. (**a**) The interactive chart for the scatter plot of different statistical scores, which depicts the iHS distribution of genetic hitchhiking region surrounding the *LCT* gene in the CEU population. (**b**) The worldwide allele frequency map of a genetic variant rs10188066 and selected population is marked with a red outline. Derived allele frequency is marked with blue color and ancestral allele frequency is marked with red color in each pie chart. (**c**) The summary table of important statistical terms for selected variant. (**d**) The three tabs records detailed information about selected variant including variant attributes, selective scores, literature evidence, mapped gene, GWAS information, cross population selective signals and comprehensive variant annotations from the external browser. (**e**) The searchable table collected literature-based positive selections in the human population.

dbPSHP uses a series of user-friendly interfaces to display the results, which not only efficiently present the query result but also facilitate the knowledge findings. The top left panel of the result page consists of three tabs. It first provides a scatter plot drawing the distribution of selective scores in the query region. A user can switch among different attributes by changing the select box. In this function, dbPSHP only returns the loci containing the selected attribute. The chart can be clicked, zoomed and is highly interactive with summary table below (Figure 1a). Besides, dbPSHP uses Google Map KML to generate the allele frequency map for all populations of selected SNP data set on a global Google Map, which provides an intuitive view for the allele distribution worldwide. The current population will be highlighted by a red outline (Figure 1b). A user can click each pie chart to get detailed information about the population in this map. dbPSHP also customizes related tracks in the UCSC Genome Browser, and a user can check it in the last internal tab of this panel.

Below the abovementioned panel, dbPSHP offers a summary table that extracts some important attributes for selected variants. Each row on the table can be clicked and is interactive with the above scatter plot (Figure 1c). The right panel in the result page has three tabs that show detailed information about a selected variant. The ‘dbPSHP Information’ tab lists the important attributes related to positive selection and reports the information of a published selective region as well as previous GWAS results recorded in GWASdb (30) (Figure 1d). ‘Cross Population’ tab records cross-population scores between queried population and each of the other populations by several statistical measurements including ΔDAF, *F*_ST_, XP-EHH and XP-CLR. To facilitate the identification of driver mutation in the investigated genetic hitchhiking region, a particular tab ‘Variant Annotation’ connects current variants to a comprehensive annotation browser SNVrap (31).

To benefit from efficient storage and simplify querying from the client environment, we encapsulated all selective attributes into a VCF INFO field and created an indexed VCF compressed file for each population using Tabix (32). Users can extract information by vcftools (33) for further process. dbPSHP also hosts a FTP server which contains compressed files and curation data for downloading. Because the full database is relatively large, we further provided RESTful style of Web Services for instant retrieving of interested regions by different interfaces.

dbPSHP hosts a repository with collected literature-based loci with positively selected signals as well as their effects (Figure 1e). Users can query the records by text-free description such as ‘rs16891982’, ‘Pigmentation’, ‘LCT’ and ‘chr6:148734174-149732519’. Besides, dbPSHP also accepts the submission of newly discovered positive selections, which will be added into dbPSHP after double checking.

## DISSCUSSION

dbPSHP is a database that systematically collects reported function-related regions/genes under recent positive selection in the human population. Our manually curated database will be frequently updated. dbPSHP further compiles a comprehensive resource that uses 15 evolutionary/statistical terms for the world-wide populations from the HapMap 3 and 1000 Genomes Project. Users can conveniently retrieve the information in either website or client by flexible queries. A set of visualization pages provides extensive views for intuitive identification of different selective signals. We believe this resource will help researchers efficiently identify, visualize and validate putative positively selected loci, as well as the causal mutation, in human evolution, and to further discover the mechanism behind these natural selections.

The statistical scores used in the database have been widely used to efficiently identify the genetic signatures of natural selection and accelerate follow-up downstream functional study. The imprint of evolutionary selection on ENCODE regulatory elements have been substantially studied, and many positive or negative selection regions are found to be functionally relevant (34). As the genome-wide association studies (GWAS) and the emerging whole genome sequencing studies (WGS) are discovering a huge number of disease associated genetic variants, future studies will be focused on the functional validation of these genetic variants, where human evolution is an essential part. Systematic evaluation of the selection attributes of associated genetic variants detected by GWAS may facilitate the finding of true causal loci for complex traits of specific population (35). Many traits/diseases associated-SNPs (30) expressed population-specific alleles as a result of different natural selection patterns across the population by polygenic adaptation (36–38). Using the evolutionary spectrum based on SNPs data and comprehensive genomic data, researchers have successfully identified many locally adapted genes or loci under environmental selection (39–41).

In addition, tracking the natural selection between human and other species can also promote functional implications of positively selected loci. With high-coverage genome data, researchers successfully identified lots of orthologous genes under positive selection across mammalian or primate genomes (42,43). Apart from genes, many other genomic elements have also been revealed under positive evolutional selection according to inter-species investigation, which include transcription factor binding sites (44), enhancers (45), non-coding DNAs (46) and transposable element-derived fragments (47). These results can efficiently benefit the functional interpretation of shared genomic elements driven by similar adaptive forces between species. Also, it will greatly facilitate the finding of genomic loci, which are selected uniquely during recent human evolution.

It is noticeable that there are many strategies to detect the true selective outliers from the background. For example, the normal range of *F*_ST_ lies between 0–1, but negative values may indicate sampling error, which should be excluded in the following procedure. Traditionally, the empirical *F*_ST_ *P*-value can be obtained by fitting to genome wide empirical distributions of *F*_ST_, which are generated from SNPs data. To eliminate the false positive loci from genome scans when using *F*_ST_, a researcher proposed a hierarchical island model comparing with a simple island model (48). Besides, simulated DNA sequence can also be used to generate neutral distributions to test the probability of a *F*_ST_ without ascertainment biases (49). Another widely used approach is to identify the candidates of selection regions from iHS. It is suggested that raw iHS need to be binned by defined genetic distance first and the variant with derived allele frequency <5% should be removed. Then, a sliding window of 50 SNPs is applied to compute the percentage of SNPs with |iHS| >2. The same strategy is also usually adopted in the processing of XP-EHH. Therefore, many raw statistical values in our database should be rightly fitted to the desired context when distinguishing true signals from noises. Some factors could also influence the sensitivity and specificity of positive selection detection methods. For example, genetic drift can drive a derived allele to fixation, which should be distinguished from selection. Ratnakumar *et al.* proposed that genes identified as targets of positive selection had a significant tendency to exhibit the genomic signature of GC-biased gene conversion (50). We also identified that the nucleotide substitutions ratio (W->S/S->W) in recent selection dataset of three populations was significantly elevated than that in all genes (Supplementary Methods). Recently, a study showed that pervasive genetic hitchhiking drives the simultaneous emergence of mutational cohorts in yeast (51), and the loss-of-function mutations can contribute to the adaptation of bacteria by rewiring a regulatory or a metabolic network (52). These findings also pointed out new strategies to track the positive selection signals in human populations.

## SUPPLEMENTARY DATA

Supplementary Data are available at NAR Online, including [53–58].

## FUNDING

Research Grants Council [781511M] of Hong Kong and NSFC [91229105] of China. Funding for open access charge: NSFC [91229105] of China.

*Conflict of interest statement*. None declared.
